# An intelligent artificial throat with sound-sensing ability based on laser induced graphene

**DOI:** 10.1038/ncomms14579

**Published:** 2017-02-24

**Authors:** Lu-Qi Tao, He Tian, Ying Liu, Zhen-Yi Ju, Yu Pang, Yuan-Quan Chen, Dan-Yang Wang, Xiang-Guang Tian, Jun-Chao Yan, Ning-Qin Deng, Yi Yang, Tian-Ling Ren

**Affiliations:** 1 Institute of Microelectronics and Tsinghua National Laboratory for Information Science and Technology (TNList), Tsinghua University, Beijing 100084, China; 2 Ming Hsieh Department of Electrical Engineering, University of Southern California, Los Angeles, California 90089, USA

## Abstract

Traditional sound sources and sound detectors are usually independent and discrete in the human hearing range. To minimize the device size and integrate it with wearable electronics, there is an urgent requirement of realizing the functional integration of generating and detecting sound in a single device. Here we show an intelligent laser-induced graphene artificial throat, which can not only generate sound but also detect sound in a single device. More importantly, the intelligent artificial throat will significantly assist for the disabled, because the simple throat vibrations such as hum, cough and scream with different intensity or frequency from a mute person can be detected and converted into controllable sounds. Furthermore, the laser-induced graphene artificial throat has the advantage of one-step fabrication, high efficiency, excellent flexibility and low cost, and it will open practical applications in voice control, wearable electronics and many other areas.

## Abstract (web summary)

The functional integration of sound generation and detection on a single device is required to assist mute people. Here, the authors demonstrate a graphene-based artificial throat capable of detecting and converting diverse throat vibrations into meaningful sound within a single device.

Owing to diseases and unexpected accidents, a lot of people in this world are not able to speak out with languages. Some technologies have been developed to help them express themselves in other ways. For example, eye tracking technology is developed to convert the eye movements into language expressions. They can use their eyes to focus on their wanted characters^1^,^2^. However, these technologies are not widely applied due to their complexities and extreme costs. In fact, many mute people have the ability to produce some kind of sounds such as a cough, hum or scream, which do not have clear meaning to other people. It is of great significance to develop an easy-to-use artificial throat that can convert the sound with unclear meaning into controllable and precise sound in languages. It means that the artificial throat should have the capabilities of both detecting and generating sound. However, acoustic transducers that can achieve this usually work with narrow bandwidth in the ultrasound range^3^ or under water^4^,^5^. Traditional sound sources and detectors are usually isolated from each other and are discrete within the human hearing range. Besides, they have the drawbacks of resonant peak and poor flexibility, which are not suitable for wearable applications.

The research of nanomaterials and nanotechnologies are contributing to the development of novel acoustic devices based on new mechanisms. For example, thermoacoustic sound sources based on graphene^6^,^7^,^8^,^9^,^10^,^11^,^12^, carbon nanotube^13^,^14^, silver nanowires^15^ and indium tin oxide^16^ have attracted a lot of attentions due to their high transparency, low heat capacity, broad frequency and excellent flexibility. However, the complex fabrication process, low yield and high cost will restrict the commercial and practical applications of thermoacoustic sources. Except novel sound sources, some nanomaterial-based novel sound detectors^17^,^18^,^19^,^20^,^21^,^22^ have been developed based on the piezoresistive effect. These novel sound detectors have responses towards the movement of throat. However, the responses are indistinctive and there is still a long way to make them for practical applications. Most importantly, the different working mechanisms of thermoacoustic sound sources and piezoresistive sound detectors make it hard to realize the functional integration of the sound source and detector in a single device. On one hand, the subsistent sound detectors based on piezoresistive materials^18^,^21^,^22^,^23^ cannot work as sound sources. Most of these sensors were encapsulated in the elastic polymers such as Ecoflex^20^ or polydimethylsiloxane^22^, which made it impossible to emit Joule heating into the air so that they cannot work as sound sources. The rest sensors^18^,^21^,^23^ have very high original resistances (up to MΩ); therefore, little Joule Heating could be generated. Previous reported sound detectors cannot work as sound sources; thus, these kinds of sound detectors can be replaced by conventional microphones. On the other hand, the thermal sound sources that were developed previously^11^ cannot work as sound detectors, because the gauge factor of laser scribed graphene is only 0.11 (ref. 24), which means it has a poor sensitivity and is hard to work as a sound detector; thus, these kinds of sound sources can be replaced by conventional speakers. However, none of the previously demonstrated nanomaterial-based acoustic device can have both sound-emitting and -detecting ability in hearing range, and this kind of multifunctional device would not be replaced by any conventional devices. Nevertheless, the functional integration of generating and detecting hearable sound in a single device has not been explored until now.

It is known that laser scribing technology has been demonstrated as an efficient method to fabricate graphene-based devices^25^,^26^,^27^,^28^,^29^,^30^. Some graphene devices based on laser scribing technology has been developed^11^,^27^, which shows advantages of large scale and customized pattern. However, the preparation of graphene oxide and the spin coating process still cause a waste of time. Lin *et al*.^31^ developed a method of converting polyimide (PI) into porous graphene films via one-step process. This method is further applied to develop supercapacitor energy storage devices^32^ and flexible strain sensors^33^,^34^. The high thermal conductivity and low heat capacity of laser induced graphene (LIG) is ideal for thermoacoustic sound sources. Besides, the porous structure will have a sensitive response towards weak vibrations, which is suitable for sound detection. Therefore, the simple and one-step fabrication will be suitable for the realization of this novel artificial throat.

Herein, we develop a one-step, wearable and low-cost LIG artificial throat by direct laser writing of PI and it shows good performance in generating and detecting sounds. This LIG artificial throat has completely different working mechanism compared with conventional acoustic transducers, which usually utilize piezoelectric effect and inverse piezoelectric effect^35^,^36^,^37^. When working as a sound source, LIG artificial throat can generate wide-band sound with frequency from 100 Hz to 40 kHz. A thinner LIG will produce higher sound pressure level (SPL). When working as a sound detector, LIG artificial throat shows unique responses towards different kinds of sounds and throat vibration modes. LIG can recognize cough, hum and scream with different tones and volumes. Besides, it also has the capability of recognizing words and sentences. Benefiting from its capability of generating and detecting sounds, the intelligent LIG artificial throat will significantly assist for the disabled. The throat vibration with different volume and frequency can be converted into controllable and predesigned sounds. Furthermore, the LIG artificial throat has the advantage of one-step process, high efficiency, excellent flexibility and low cost. The LIG can be acquired by using a portable and low-power laser platform, which will reduce the risk under operation. The LIG artificial throat has promising applications in the fields including voice control, wearable electronics and many others.

## Results

### Fabrication and characterization of LIG artificial throat

**Figure 1:**
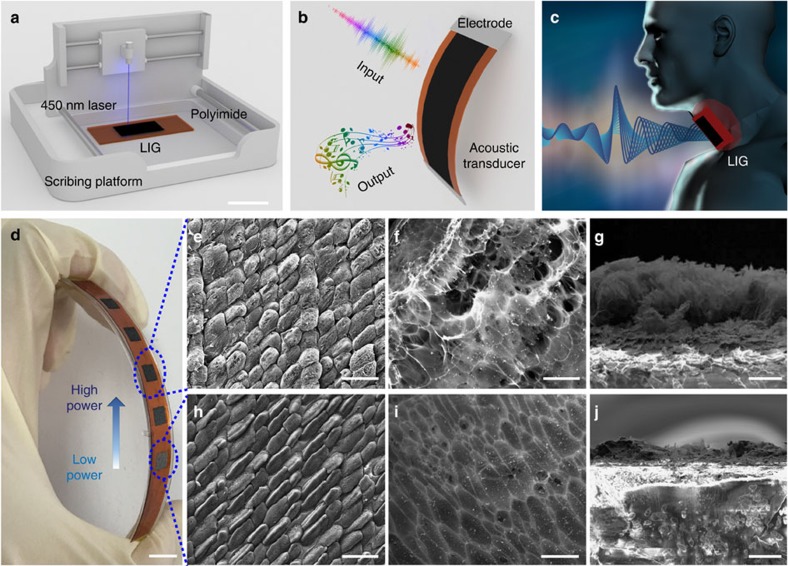
Schematic illustration of the fabrication process and the morphology of LIG. (**a**) One-step fabrication process of LIG. PI is directly converted into LIG by the irradiation of the 450 nm laser. Scale bar, 2.5 cm. (**b**) LIG has the ability of emitting and detecting sound in one device. (**c**) The artificial throat can detect the movement of throat and generate controllable sound, respectively. (**d**) Six LIG samples produced by 450 nm laser with different power ranging from 20 to 350 mW. Scale bar, 5 mm. (**e**) The morphology of LIG sample produced at 290 mW under scanning electron microscopy. Scale bar, 150 μm. (**f**) The morphology of LIG sample produced at 290 mW under high magnification. Scale bar, 5 μm. (**g**) Cross-sectional view of LIG sample produced at 290 mW. Scale bar, 12.5 μm. (**h**) The morphology of LIG sample produced at 125 mW under scanning electron microscopy. Scale bar, 150 μm. (**i**) The morphology of LIG sample produced at 125 mW under high magnification. Scale bar, 5 μm. (**j**) Cross-sectional view of LIG sample produced at 125 mW. Scale bar, 12.5 μm.

Laser direct writing technology promotes the fast growth of porous graphene, and a low-cost and portable laser platform is chosen in this work (Supplementary Fig. 1). Figure 1a shows the one-step process of the LIG. The PI film is located under the 450 nm laser and converted into LIG by direct irradiation of laser. The *X*–*Y* directional motors control the movement of the laser so that a predesigned pattern can be irradiated at precise locations (see the Methods section for details). For instance, Tsinghua University's logo and a 6 cm × 4 cm rectangle are imported into the computer control software, and the same patterns are generated on the PI film by converting PI into graphene (Supplementary Fig. 2). Here, a simple rectangle LIG is produced to work as an artificial throat. As illustrated in Fig. 1b, the artificial throat has the integrated functions of emitting and detecting sounds. When an AC voltage is applied on the device, the periodic joule heat will cause the expansion of air, resulting in sound waves. When a low bias voltage is applied on the device, the vibration of throat cords will cause the change of the device's resistance, resulting in a fluctuation of the current. Therefore, the devices can work as sound source and detector at the same time. The working principle of the LIG artificial throat is shown in Fig. 1c. A cough, hum or scream will cause the vibration of throat cords, which can be detected by the LIG artificial throat, and then LIG artificial throat will generate controllable sounds accordingly. Therefore, the LIG artificial throat can realize the conversion from meaningless sounds to controllable and predesigned sounds.

The photograph of LIG generated at different laser power *P* ranging from 20 to 350 mW is shown in Fig. 1d. The most bottom LIG is irradiated at *P*=20 mW and no obvious LIG is observed (as shown in Supplementary Fig. 3). The second one and the fourth one from the bottom, which are generated at *P*=125 mW and *P*=290 mW, respectively, are chosen to show their scanning electron microscope images because of their typical morphologies. As shown in Fig. 1e–j, it can be noticed that ridgy lines are formed orderly along the scanning trace of laser from up to down. The line width is around 100 μm, which is similar to the focus spot size of the laser. Morphological differences can be clearly observed with the increasing of laser power. More pores are produced at *P*=290 mW. From the high-magnification image, the differences between the structures become more distinctive. A thin layer of carbon sheet with porous polygon appears at *P*=125 mW and more porous and irregular structure is produced at *P*=290 mW, because local high temperature during laser heating conduct the pyrolysis of PI^38^,^39^, breaking the C–O, C=O and N–C bonds, and causing the venting of some carbonaceous and nitric gases. From the side view, we can notice that the thickness increases from ∼10 to ∼40 μm at *P*=125 mW and *P*=290 mW, respectively. The structure generated at 350 mW (Supplementary Fig. 4) becomes more porous and thicker.

The Raman spectrums of PI film and samples generated at *P*=125 mW and *P*=350 mW are performed for further investigation (Supplementary Fig. 5). The spectrums obtained at *P*=125 mW and *P*=350 mW show the similar characteristics with a *D*, *G* and 2*D* peak at 1,350, 1,580 and 2,700 cm^−1^ respectively. The Raman spectrum is clearly different from amorphous carbon, proving the existence of randomly graphene stacks. With the increasing of the laser power, the intensity of *D* peak increases, indicating that high-power laser makes the structure more defective and disordered. Moreover, the high-resolution transmission electron microscope image presents the lattice fringes with an interspace of ∼3.4 Å, corresponding to the interplanar spacing of (002) plane in graphic materials (Supplementary Fig. 6).

### LIG artificial throat working as a sound source

**Figure 2:**
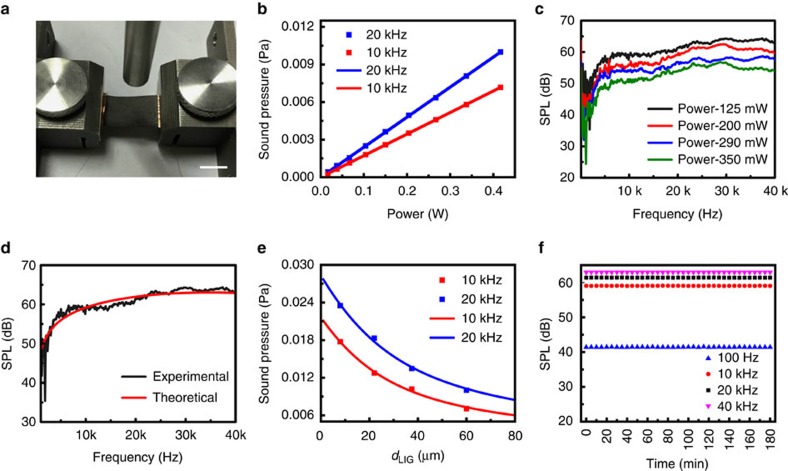
The performance of emitting sound. (**a**) The LIG is clamped under a commercial microphone to test the performance of emitting sound. Scale bar, 1 cm. (**b**) The plot of the SP versus the input power at 10 and 20 kHz. The square is the experimental result and the line is the theoretical result. (**c**) The output SPL versus the frequency of LIG generated by the laser with different power. The four curves are normalized with the input power of 1 W. (**d**) The SPL versus the frequency showing that the model agrees well with experimental results. (**e**) The plot of the SP versus the thickness of LIG at 10 and 20 kHz. The square is the experimental result and the line is the theoretical result. (**f**) The stability of output SPL over time.

Four samples of LIG artificial throats, which are produced at different laser powers, are used to test the performance of emitting sound. The area of the LIG is around 1 × 2 cm^2^. The laser powers are 125, 200, 290 and 350 mW. The average thickness of LIG is 8, 22, 38 and 60 μm. The LIG artificial throat is clamped under a commercial microphone as shown in Fig. 2a. The distance between LIG and microphone is 2.5 cm (see the Methods section for details). As shown in Fig. 2b, the output SP of LIG (generated under 125 mW) increases with the input power and the LIG has a higher efficiency at 20 kHz. The fitting line shows that the SP has a linear relation with the input power. The relationships between SPL and the frequency of different samples are demonstrated in Fig. 2c. The experimental results are normalized with the same power (1 W). It is noticed that the SPL becomes lower with the increasing of laser power and the thickness of LIG. A similar phenomenon was found in the work by Tian *et al*.^6^.

The theoretical model of thermal acoustic is first built by Arnold and Crandall^40^. In recent years, a new model of the thermal acoustic effect is proposed based on the energy conservation^41^, which makes it easy to analyse and calculate. The core of thermal acoustic is the propagation of the heat. SP is decided by the thermal energy diffused into air:^42^


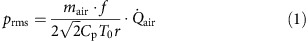


where *m*_air_ is the molecular weight of air, *f* is the frequency of the acoustic, *Q*_air_ is the thermal energy diffused into air, *C*_p_ is the heat capacity at constant pressure, *T*_0_ is the room temperature and *r* is the measuring distance from the source.

The thermal energy is calculated as:^41^


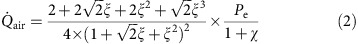


where 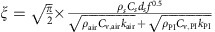, 
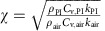
, and 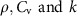 are the density, heat capacity at constant pressure and conductivity of each material, respectively. *d*_s_ is the thickness of the LIG and *P*_e_ is the input power. From equation (2), we can get the SPL as a function of frequency and the thickness of LIG.

Figure 2d is the comparison of theoretical curve and the experimental results. The experimental analysis matches well with the theory model. Figure 2e shows the experimental data and theoretical curve as a function of the thickness of the LIG under the frequency of 10 and 20 kHz. The four samples with the thickness of 8, 22, 38 and 60 μm would produce the sounds with the SP of 0.01775, 0.01276, 0.01019 and 0.00706 Pa at the frequency of 10 kHz and 0.0235, 0.0183, 0.01346 and 0.01 Pa at the frequency of 20 kHz, respectively. The theoretical curve matches well with the experimental results. As the decrease of the thickness, the leakage of thermal energy will be reduced and more thermal energy will be propagated into the air, implying that a thinner LIG will generate a higher SP. The experimental data fit well with the theoretical model. The working temperature is tested by a thermal infrared camera (Fluke Ti 200). The working temperature are 23.7 °C with 0.4 W input power and below 29 °C even with 1.225 W input power, which are acceptable when attached to the skin (Supplementary Fig. 7). The SPL is measured for a long time to investigate the stability of LIG. SPL at 100 Hz, 10 kHz, 20 kHz and 40 kHz are shown in Fig. 2f. There are no signs of degradation or changes in the device performance in 3 h.

### LIG artificial throat working as a sound detector

**Figure 3:**
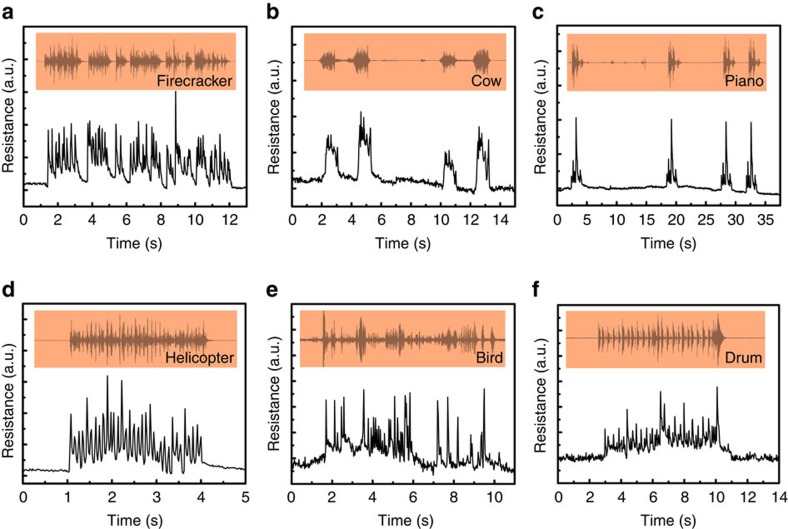
Responses towards different audios from a loudspeaker. The LIG is placed 3 cm away from the loudspeaker. The orange insets above indicate the sound wave profiles of the original audios. Relative resistance changes show almost synchronous response to profiles of the original audios when the loudspeaker plays the audio of (**a**) firecrackers, (**b**) a cow, (**c**) a piano, (**d**) a helicopter, (**e**) a bird and (**f**) a drum.

Except for emitting sound, the LIG artificial throat also has excellent responses when detecting sound. PI with different thickness will have different performance of recognition. A 25 μm-thick PI is chosen to generate LIG because of the obvious resistance change compared to the PI with the thickness of 75 and 180 μm (Supplementary Fig. 8). The sensitivity is high enough to detect SP produced by a loudspeaker. The 25 μm PI–LIG is fixed by two clamps free-standingly and the loudspeaker is placed 3 cm away from the artificial throat. The audio tests with six kinds of audios including firecracker, cow, piano, helicopter, bird and drum are performed. The results are shown in Fig. 3. Although the sampling frequency of our artificial throat is 100 Hz, which is far lower than the frequency of sound, we can still notice that the responses of the transducer are well synchronous to these original audio signals (orange parts). Especially, the characteristic peaks are retained and reflected with high fidelity. Besides, the volume of loudspeaker has a great effect on the amplitude of the signal. With the volume increases, the vibration will be strengthened, causing a more obvious change of resistance (Supplementary Fig. 8). The film is clamped and bent under the force (Supplementary Fig. 9). The curvature radius is 1.27 cm and the stress is 0.07 N. The bending degree is higher than the vibration degree, which is caused when speaking. The testing results after 0 cycle, 1,000 cycles and 10,000 cycles are shown in Supplementary Fig. 9. We can see the device shows excellent durability under high strain.

**Figure 4:**
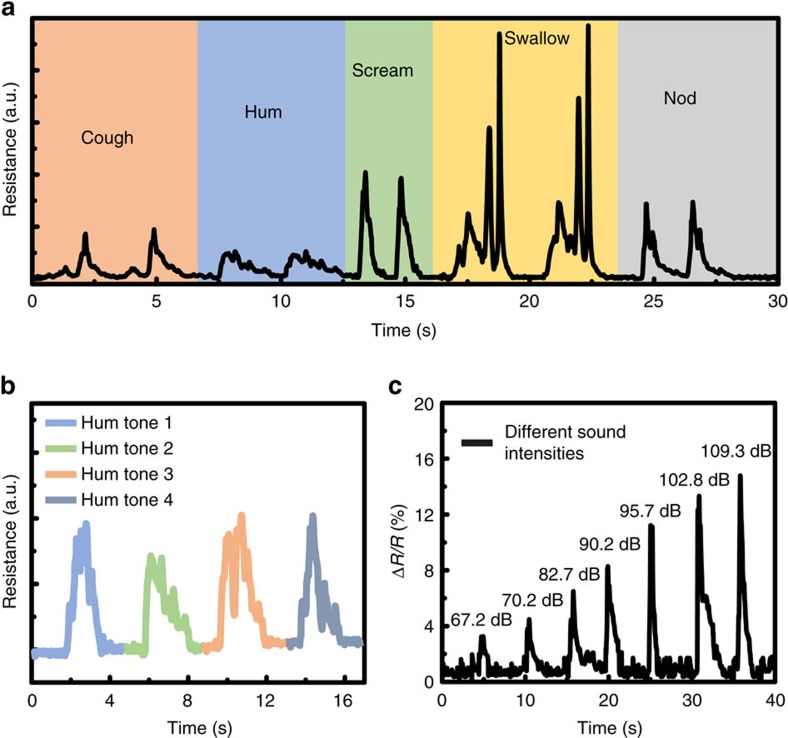
Responses towards different kinds of throat vibrations. (**a**) The LIG's resistance changes towards the throat vibrations of the tester who makes two successive coughs, hums, screams, swallowing and nods. (**b**) The LIG's resistance change caused by four different kinds of hum tones and the hum tone 2 is same with the hum in **a**. (**c**) The relative resistance change of LIG increases with the increase of the sound intensities of the hum.

After identifying some kinds of audio clearly, LIG throat is used to detect the vibration of throat cords. As shown in Fig. 4a, the tester makes two successive coughs, hums and screams, and then the tester does swallowing and nod actions in two times. The repeatability of the detection is excellent according to the two-time successive testing. Besides, the swallowing and the nod can cause the muscle movement, which can also result in the change of the resistance. Fortunately, the waveforms of these kinds of muscle movements also have recognizable characteristics. Different movement has its unique characteristic waveform as shown in Fig. 4a; thus, we can get the useful waveforms by relying on the pattern recognition and machine learning. The interference by some other activities can be recognized and eliminated by training many times in advance. Then, the tester makes the hums with four different tones as shown in Fig. 4b; we can see that different tones also have different responses, increasing the variety of the ‘language' of mute persons. Especially, the hum tone 2 is same with the hum in Fig. 4a. Furthermore, as shown in Fig. 4c, the resistance increases as the sound intensity increases, resulting from the increase of the mechanical vibration of throat cords.

When the device is attached on the throat, it can detect both SP and throat vibration. An experiment is performed to compare whether the mechanical vibration of throat cords or the SP contributes most to the relative resistance change of the LIG artificial throat. The LIG is attached on the throat of the tester and the tester makes some hums with different volumes, and these sounds are recorded and played by a loudspeaker. The LIG then is placed 3 cm away from the loudspeaker, to acquire the signal induced by pure SP. According to Supplementary Fig. 10, the relative resistance change of the device placed 3 cm away from the loudspeaker is only 0.5% when the loudspeaker plays a sound of 90 dB, which means the pure SPL of 90 dB will cause a 0.5% relative resistance change. However, the relative resistance change of the device attached on the throat can be 8.2% when a person makes a hum of 90 dB. The relative resistance change of the device attached on the throat is 16 times larger than that caused by pure SP, which means the vibration of throat cords contributes more than the SP.

Besides, the LIG artificial throat also has the capability of voice recognition. As shown in Supplementary Fig. 11, some words including ‘Graphene', ‘Material' and ‘Industry' are pronounced by different testers including an elder, a boy, a man and a woman, respectively. The wave curves of different words in the time domain show apparently different characteristics, which is helpful to distinguish different words. Besides, the wave curves of a same word pronounced by different persons show similar but different characteristics, which can be a key factor for identity authentication by voice recognition. Furthermore, a long sentence, ‘Graphene is a carbon-based material with huge potential for industry', is spoken repeatedly by a woman for five times. The artificial throat shows excellent repeatability and reliability to work as an acoustic detector. Especially, from the magnified image of the sentence, we can notice that the three words, ‘graphene', ‘material' and ‘industry', are almost identical to the individual pronunciation by the woman. Compared with some other nanomaterial-based acoustic detectors^17^,^18^,^19^,^20^,^21^,^22^, our device demonstrates superior predominance in voice recognition because of its excellent repeatability and reliability.

### Working as sound source and sound detector simultaneously

**Figure 5:**
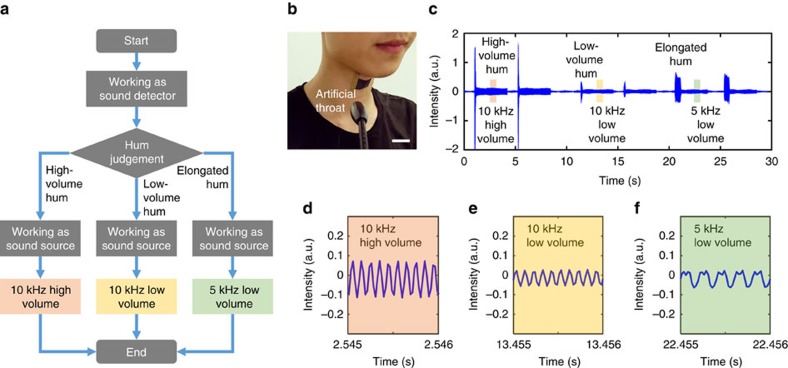
An intelligent LIG artificial throat. (**a**) The working procedure of the artificial throat. (**b**) The tester wearing the LIG artificial throat. Scale bar, 1 cm. (**c**) high-volume, low-volume and elongated tone hum are detected by LIG throat and converted into high-volume 10 kHz, low-volume 10 kHz and low-volume 5 kHz sound, respectively. (**d**) The magnified wave of high-volume 10 kHz sound. (**e**) The magnified wave of low-volume 10 kHz sound. (**f**) The magnified wave of low-volume 5 kHz sound.

The LIG artificial throat developed in this work has great potential to bring a revolution in the field of acoustic. As we know, most mute persons are born deaf and they cannot speak words. However, their throat cords can vibrate and they are capable of making noises in their own ways, which are meaningless to the normal. The intelligent LIG artificial throat is developed to transform the meaningless noise into controllable and understandable sound signals. The testers need to be trained first. When they produced a specific cough, hum or scream, we told them the corresponding meaning by gesture language. They can be accustomed to the sound intensity by multi-time repeated training. The training process is similar with the process of importing the fingerprint into iPhone. Then, these waveforms can be recognized by pattern recognition and machine learning. Besides, we set some threshold ranges in advance. The software can recognize the sound intensity when it lies on the threshold range. The accuracy of recognition can be guaranteed by the above two ways. After the training process, the different volume, frequency and last time of hum, scream or cough can be converted into specific meanings. Here we simply demonstrated the conversion from three kinds of different hums to three controllable sounds. The working principle of LIG artificial throat is shown in Fig. 5a (see the Methods section for details). It works as a sound detector at first, then the tester makes three kinds of hums to imitate a mute person and the resistance of the transducer will be changed by the throat cords vibration correspondingly. A microcontroller is used to detect the resistance change of the transducer and realize the hum judgement. After that, the transducer begins to work as a sound source and corresponding sound signal is generated. The LIG artificial throat is attached on the throat of a tester as Fig. 5b shows.

As shown in Fig. 5c, a high-volume hum, a low-volume hum and an elongated hum are pronounced twice by the tester. Correspondingly, a high-volume 10 kHz, a low-volume 10 kHz and a low-volume 5 kHz sound signal are produced. The output sound frequency is twice the input electric signal; thus, a DC bias is added to generate the fundamental tone^11^. The high-volume 10 kHz, the low-volume 10 kHz and the low-volume 5 kHz sound are generated by 10 V DC+10 V 10 kHz AC, 5 V DC+5 V 10 kHz AC and 5 V DC+5 V 5 kHz AC, respectively (Supplementary Movie 1). From the magnified images (Fig. 5d–f), the amplitude and the frequency of the output sound signals are shown as we expected. Finally, an intelligent LIG artificial throat is demonstrated to convert meaningless noises into useful sound signals with controllable frequencies and volumes. It will be possible for mute persons to express themselves with the assistance of LIG artificial throat.

## Discussion

In summary, a one-step fabricated wearable artificial throat based on LIG has been developed. The low-power laser with the wavelength of 450 nm can induce the conversion from PI to LIG. The LIG realize the functional integration of emitting and detecting sound on a single device because of its superior thermoacoustic and piezoresistive properties. As a sound source, the SPL of the LIG artificial throat has been demonstrated from 100 Hz to 40 kHz. The thickness of LIG will have an obvious influence on SPL according to our theory and a thinner LIG will generate sound with higher SPL. The LIG artificial throat has a relatively broad frequency spectrum because of resonance-free oscillations of the sound sources. Besides, as a sound detector, the LIG artificial throat can capture the mechanical vibration of throat cords with a fine repetition. It can clearly differentiate the characteristics of cough, hum and scream with different tones and volumes according to their unique waveforms. Besides, it also has the capability of voice recognition because of its outstanding mechanical properties. The intelligent LIG artificial throat will significantly assist for disabled person. The LIG artificial throat can generate volume and frequency controllable sound by detecting different kinds of imitative hum of the tester and realize the conversion from meaningless hum to controllable sound, which has significantly practical potentials. Furthermore, the one-step fabricated wearable LIG artificial throat will open widely practical applications in voice control, wearable electronics and many other areas due to its high sensitivity, excellent repetition, good flexibility and simple fabrication process.

## Methods

### Fabrication of artificial throat

A custom-designed platform equipped with a laser diode (OSRAM) with wavelength of 450 nm was used to convert PI into LIG. Figure 1a shows the schematic diagram of the mask-free process. The movement of laser was controlled by two stepper motors in *X–Y* direction. The beam size of laser and the minimum displacement of stepper motor are 100 μm. The scanning speed of laser is 8.5 mm s^−1^ and the maximum power is 500 mW. Commercial PI films (Kapon) with thickness of 25, 75, 125 and 180 μm were used as the substrate without any further treatment. After the laser irradiation, the LIG artificial throats are wired out by copper wire by using silver paste.

### Characterization

The surface morphology of the LIG is observed by a Quanta FEG 450 scanning electron microscope (FEI Inc.). Raman spectroscopy is performed using a laser with a wavelength of 532 nm (HORIBA Inc.). Transmission electron microscopic images are taken by JEM2100F (JEOL Inc.).

### Testing of artificial throat

The sound emitting testing platform consisted of a standard microphone and a dynamic signal analyser. A 1/4 inch standard microphone (Earthworks M50) was chosen to measure the SPL of the LIG artificial throat. The microphone has a very flat frequency response within 40 kHz and a high sensitivity of 31 mV Pa^−1^. The dynamic signal analyser (Agilent 35670A) was used to generate swept-frequency signals from 100 Hz to 40 kHz, to drive the LIG artificial throat and to record the value of the SPL of it. The sound-detecting testing platform consisted of a commercial loudspeaker and a digital multimeter. LIG artificial throat was placed 3 cm away from the loudspeaker. The digital multimeter (ROGOL DM3068) was used to the relationship between the resistance and the time.

### Generating and detecting sound in a single device

The LIG artificial throat was attached on the throat of a tester. The LIG artificial throat worked in two modes: detecting mode and emitting mode. During the detecting mode, a microcontroller was used to detect the amplitude and last time of the meaningless hums. When the amplitude or last time exceeded the thresholds, the microcontroller would stop detecting and the digital function generator would be applied on the LIG artificial throat for 3 s. After 3 s, the microcontroller closed the digital function generator by a relay and LIG artificial throat works in detecting mode again. Different digital function generators would be applied on the LIG artificial throat respectively according to the amplitude and last time of the meaningless noise.

### Data availability

The data that support the findings of this study are available from the corresponding author upon request.

## Additional information

**How to cite this article:** Tao, L.-Q. *et al*. An intelligent artificial throat with sound-sensing ability based on laser induced graphene. *Nat. Commun.* **8,** 14579 doi: 10.1038/ncomms14579 (2017).

**Publisher's note**: Springer Nature remains neutral with regard to jurisdictional claims in published maps and institutional affiliations.

## Supplementary Material

Supplementary Information Supplementary Figures

Supplementary Movie 1 Shows the demo of laser induced graphene working as an artificial throat (also shown in Fig. 5). From Supplementary Movie 1, the hum from the throat can be recognised by our device and our device can also convert the input hum into a controllable sound waves.
