# Supplementary material for: An intelligent artificial throat with sound-sensing ability based on laser induced graphene
Source: Nat Commun. 2017 Feb 24;8:14579. doi: 10.1038/ncomms14579 (PMC5333117; doi:10.1038/ncomms14579)
Supplement: Supplementary Information — Supplementary Figures [file ncomms14579-s1.pdf]

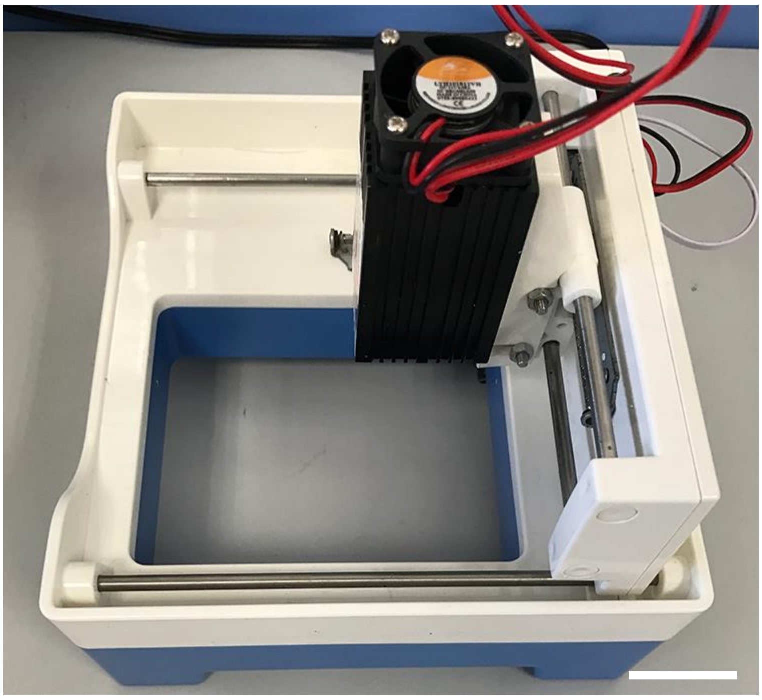

Supplementary Figure 1. The photograph of the low-cost and potable laser platform. The laser platform can realize the generation of LIG with custom pattern. The wavelength of the laser diode is 450 nm and the maximum power is 500 mW. Scale bar: 2 cm

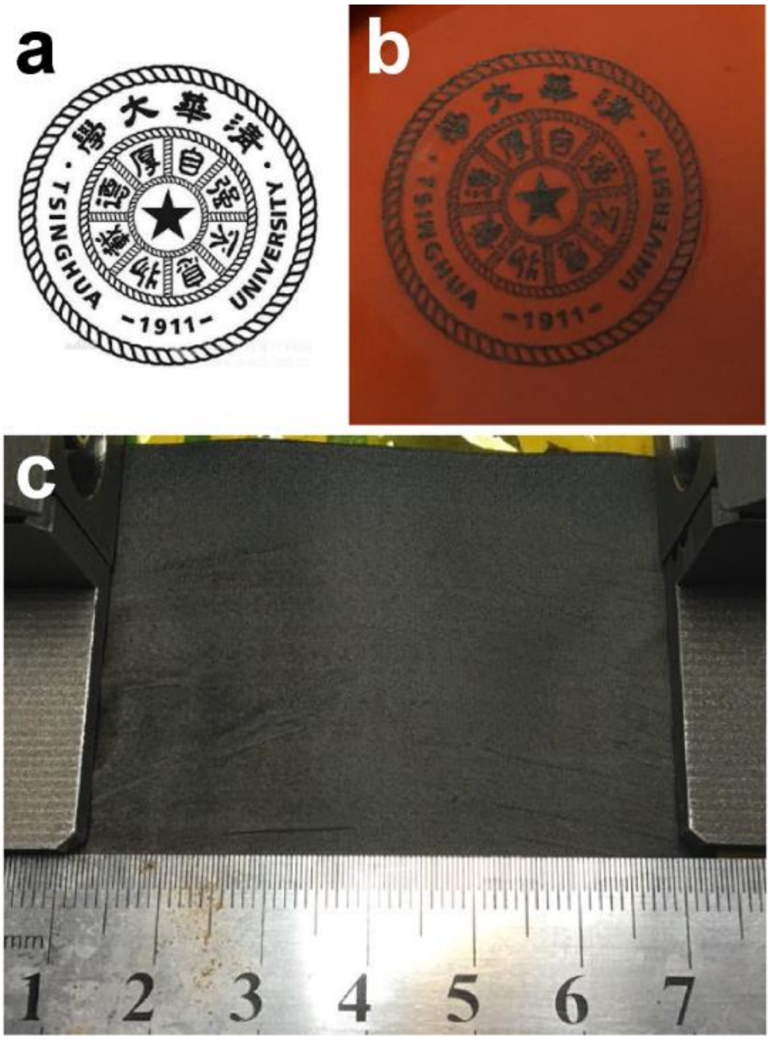

Supplementary Figure 2. The demo showing the advantage of large scale and customized pattern. **(a)** Original image of Tsinghua University's logo used for laser scribing on PI. **(b)** LIG with Tsinghua University's logo. The photo of Tsinghua University's logo is imported into the PC control software, and the same patterns are generated on the PI film. **(c)** Photograph of a 6cm×4cm-rectangle LIG.

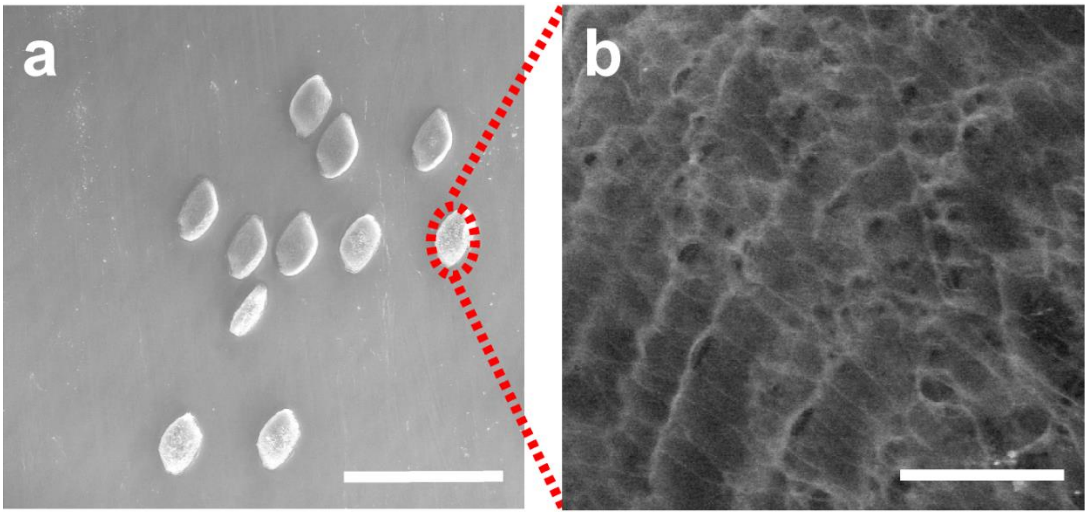

Supplementary Figure 3. The morphology of LIG sample produced at 20 mW under SEM showing a small number of LIG points are generated. **(a)** The morphology of LIG sample produced at 20 mW under SEM. Scale bar: 500 μm. **(b)** The morphology of LIG sample produced at 20 mW under high magnification. Scale bar: 15 μm.

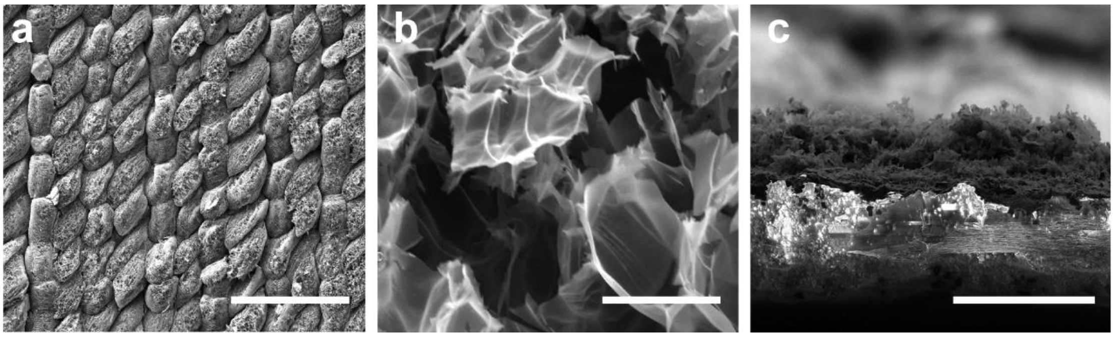

Supplementary Figure 4. The morphology of LIG sample produced at 350 mW under SEM. **(a)** The morphology of LIG sample produced at 350 mW under SEM. Scale bar: 300 μm. **(b)** The morphology of LIG sample produced at 350 mW under high magnification. Scale bar: 10 μm. **(c)** Cross-sectional view of LIG sample produced at 350 mW. Scale bar: 100 μm. Compared with the SEM image of LIG at P=125 mW and 290 mW (Fig. 1e-j), it's obvious that the structure generated at higher power will become more porous and thicker.

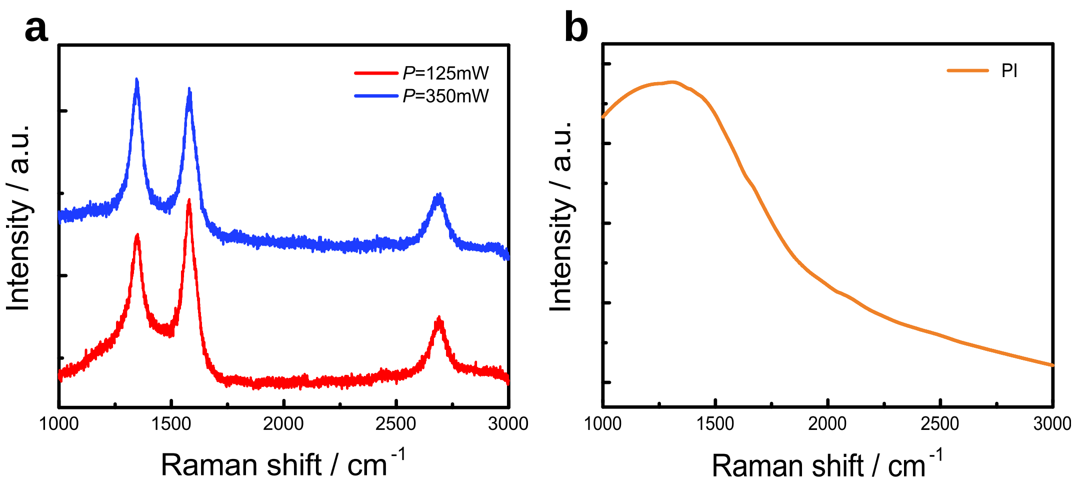

Supplementary Figure 5. The Raman spectrums of PI film and samples generated at *P*=125 mW and *P*=350 mW. **(a)** The Raman spectrum of LIG generated at the laser power of 125 mW and 350 mW. **(b)** The Raman spectrum of PI.

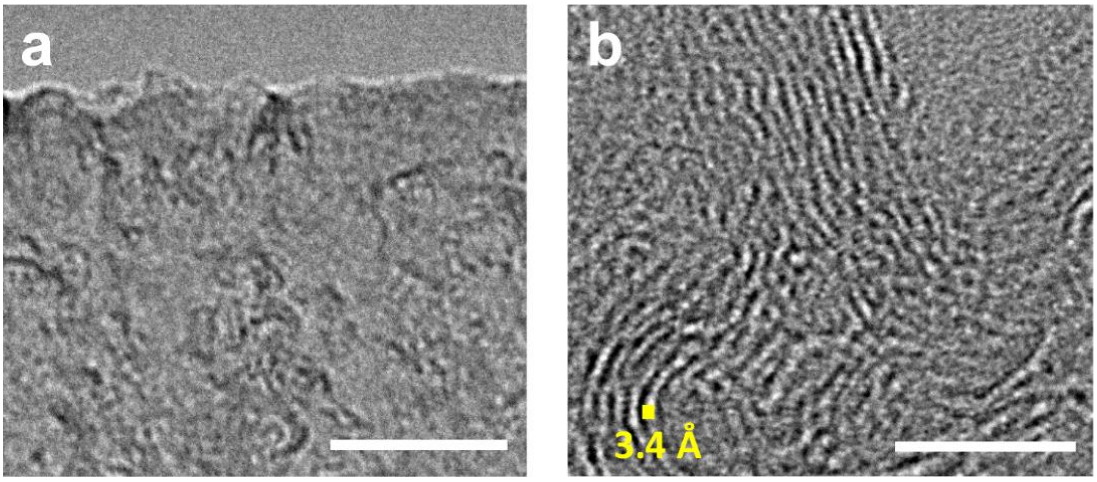

Supplementary Figure 6. The HRTEM image showing obvious lattice fringes, and the average lattice space of ~3.4 Å. **(a)** The TEM image taken at the edge of a LIG flake. Scale bar: 50 nm. **(b)** The high-resolution TEM (HRTEM) image showing the lattice space of ~3.4 Å. Scale bar: 5 nm.

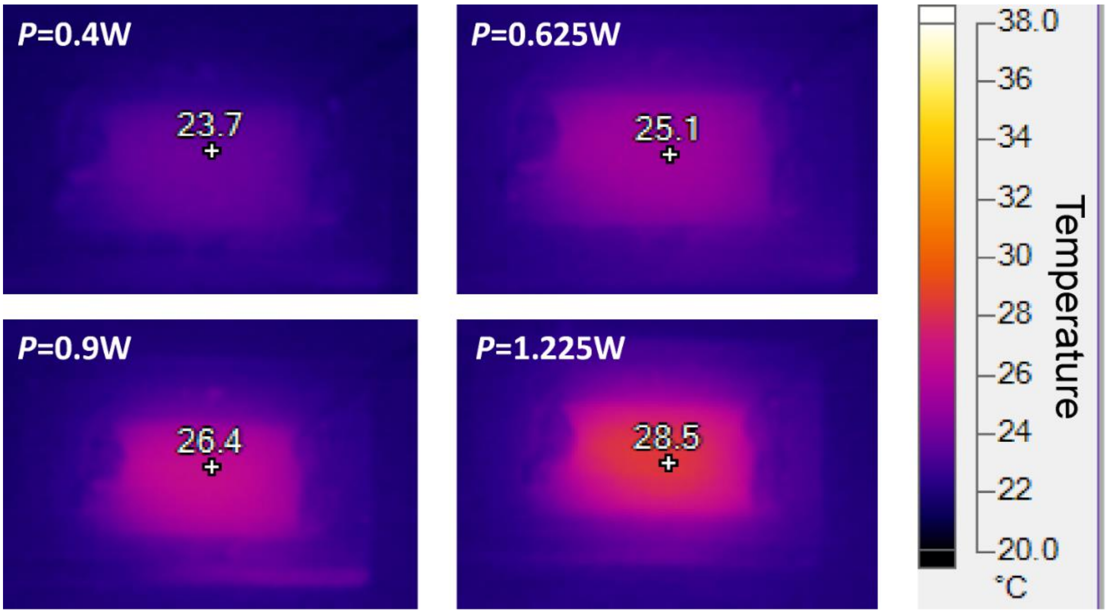

Supplementary Figure 7. The relationship between the temperature and the power. We use a thermal infrared camera (Fluke Ti200) to monitor the temperature of LIG under different input power. The working temperature are 23.7 °C with 0.4 W input power and below 29 °C even with 1.225 W input power, which are acceptable when attached to the skin.

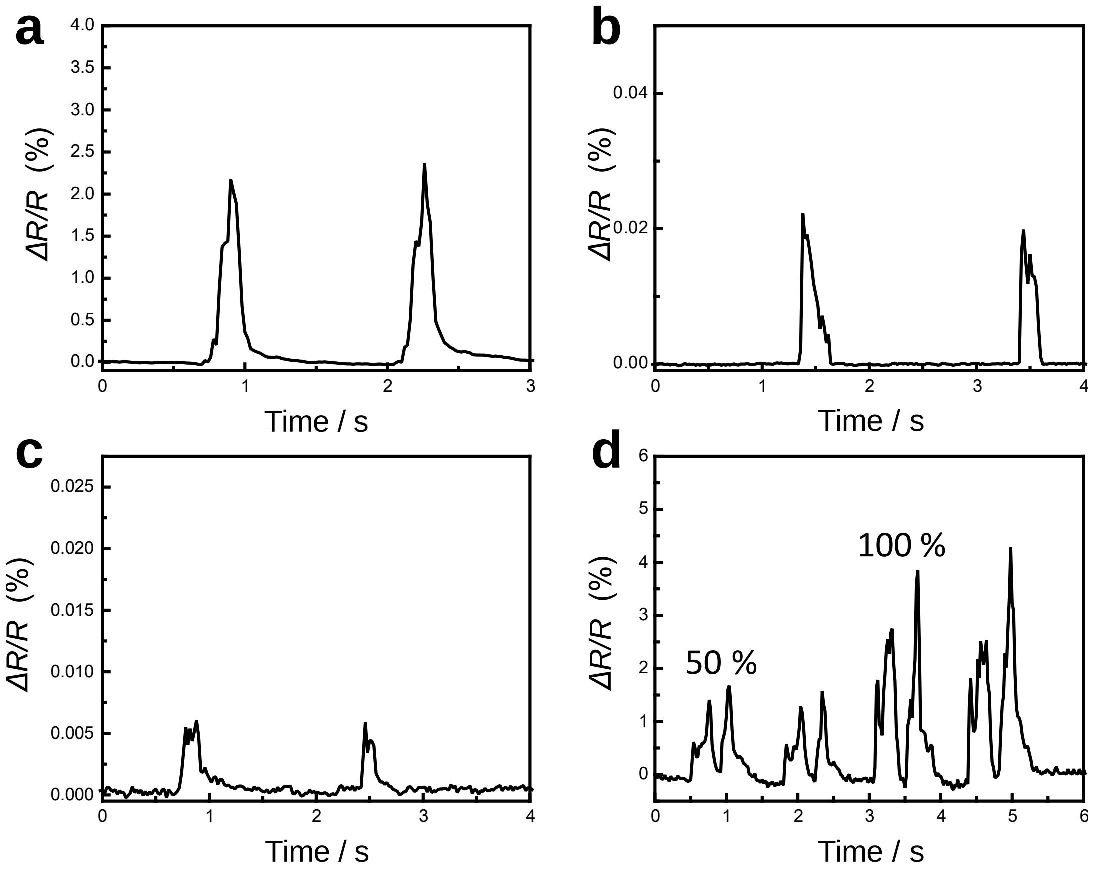

Supplementary Figure 8. The PI with different thickness showing different performance of recognition. **(a)** The resistance change of LIG generated on a PI with the thickness of 25 μm. **(b)** The resistance change of LIG generated on a PI with the thickness of 75 μm. **(c)** The resistance change of LIG generated on a PI with the thickness of 180 μm. **(d)** The resistance change under different volumes. LIG generated on the PI with the thickness of 25 μm, 75 μm and 180 μm are placed 3 cm away from the loudspeaker, and the resistance changes are almost 2%, 0.02% and 0.005% respectively. With the volume increases, the vibration will be strengthened, causing a more obvious change of resistance.

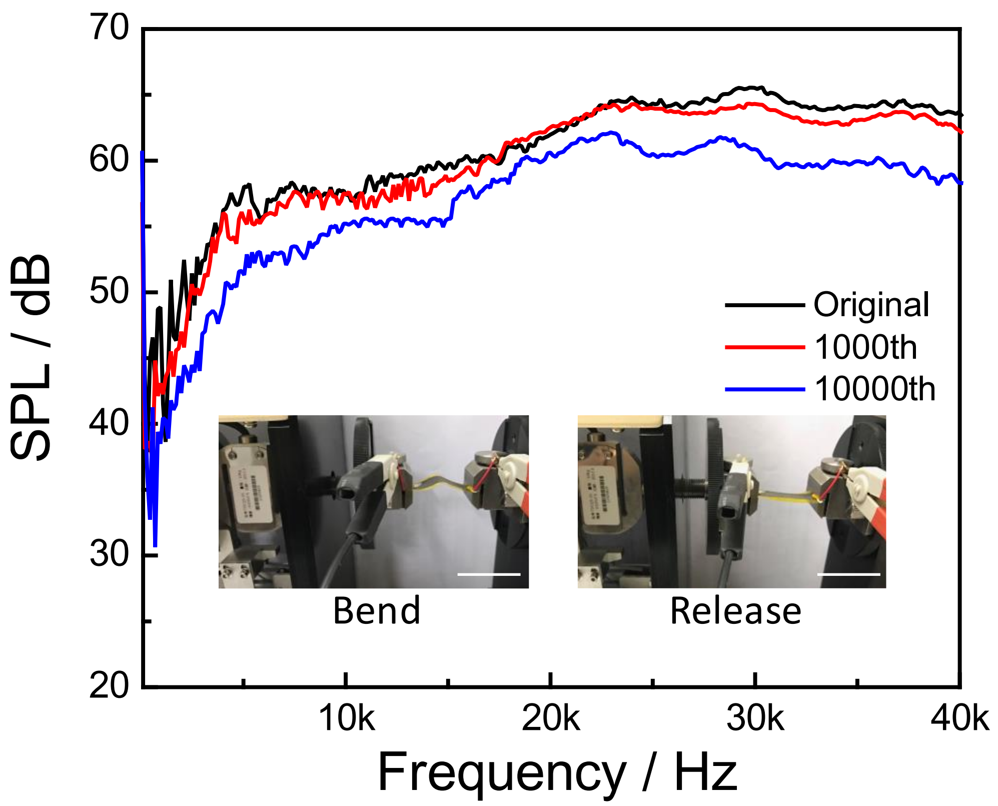

Supplementary Figure 9. The durability test of LIG up to 10000 times bending. The durability measurement is performed and the LIG is clamped and bent multi times under the force. The curvature radius is 1.27 cm and the stress is 0.07 N. The bending degree is higher than the vibration degree caused by speaking. The testing results at original state and after 1000 cycles and 10000 cycle are shown. We can see the device shows excellent durability under high strain. There is no obvious degradation of SPL up to 1000 times bending and a less than 5 dB degradation up to 10000 times bending. Scale bar: 2 cm.

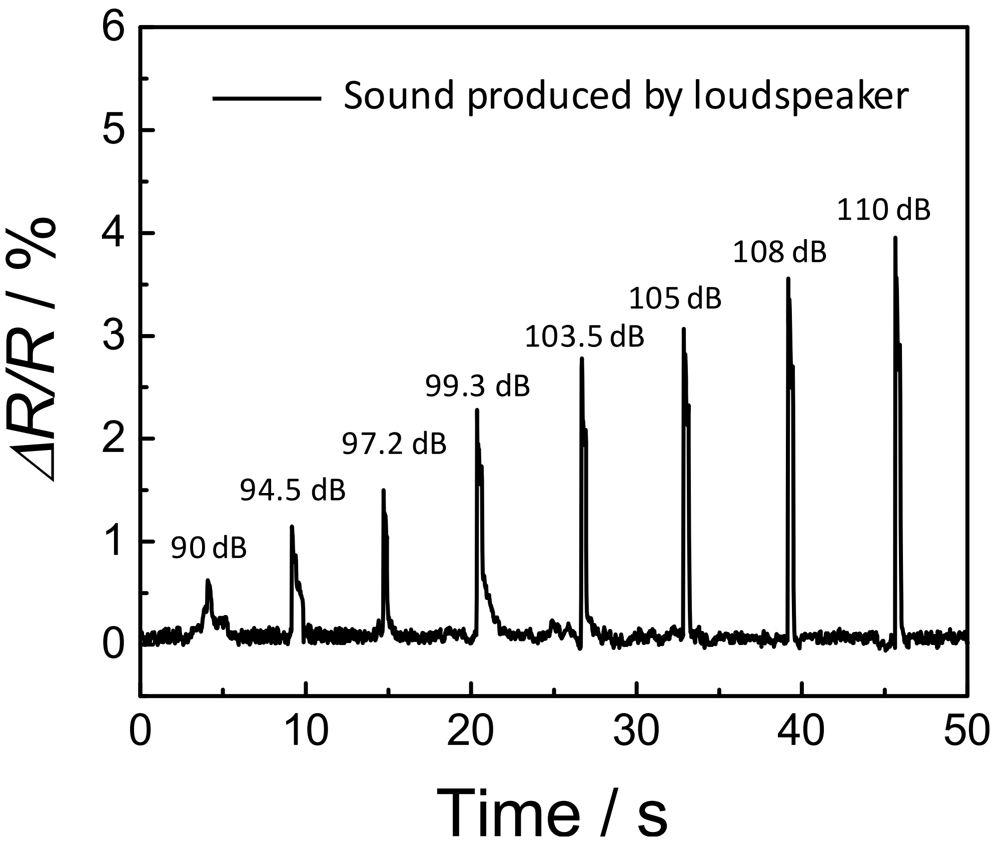

Supplementary Figure 10. The relationship between relative resistance change and sound pressure. The device was placed 3cm away from the loudspeaker and a commercial microphone was placed at the same distance to record the sound pressure level so that we can acquire the relationship between the resistance change and the sound pressure. We found that the sound pressure could cause a relative resistance change of 0.5% at 90 dB. However, the LIG attached on the throat could have a relative resistance change of 8.2% with the same sound pressure according to the figure 4c in the manuscript, which is almost 16 times larger than that induced by pure sound pressure. Therefore, we deduce that the vibration of throat cords mainly contributed the resistance change.

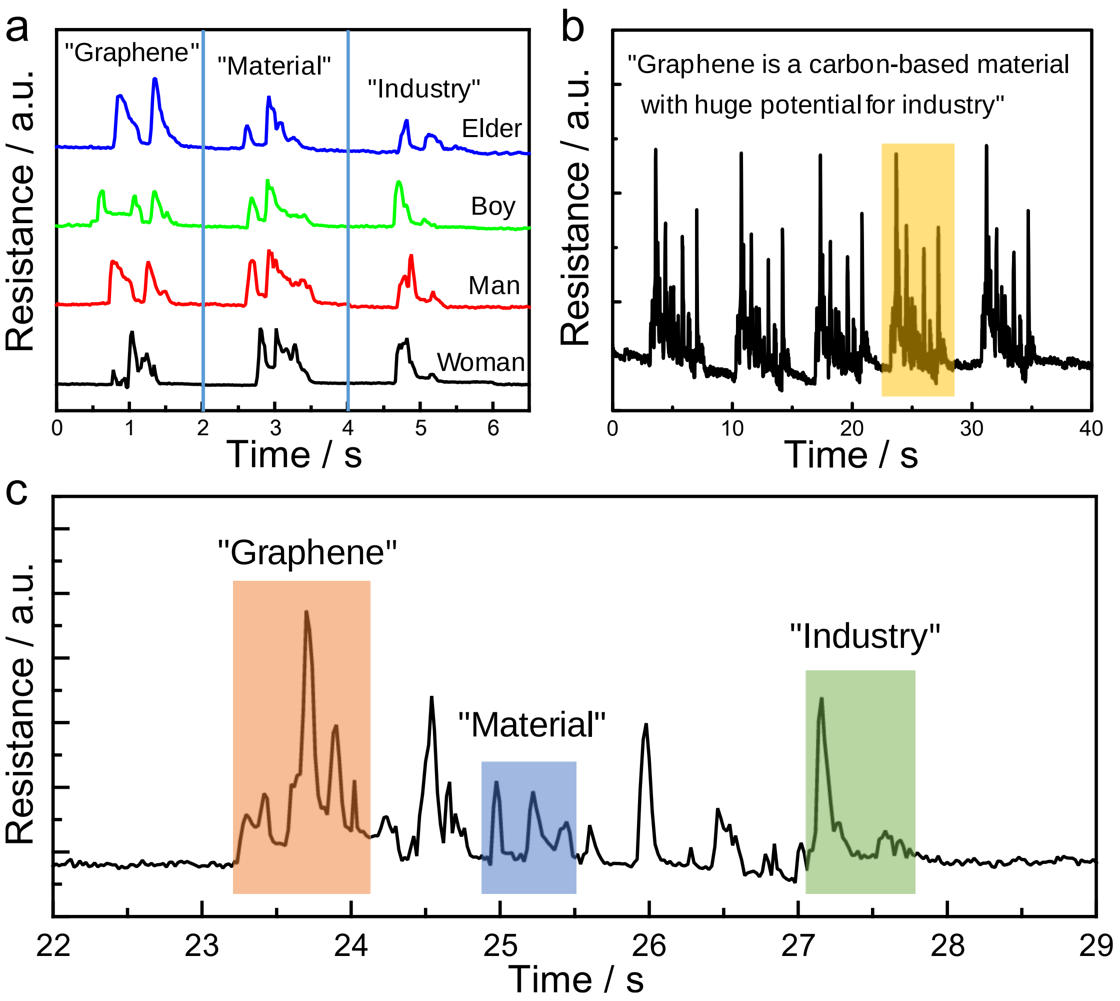

Supplementary Figure 11. The LIG artificial throat showing the capability of voice recognition. **(a)** Three words, “graphene”, “material”, and “industry”, are pronounced by an elder, a boy, a man and a woman respectively. **(b)** A long sentence “graphene is a carbon-based material with huge potential for industry” is pronounced by a woman for five times. **(c)** The magnified image of the yellow part, and “graphene” (red), “material” (blue) and “industry” (green) are marked. The wave curves of different words in the time domain show apparently different characteristics, which is helpful to distinguish different words. Besides, the wave curves of a same word pronounced by different persons show similar but different characteristics, which can be a key factor for identity authentication by voice recognition.
